# Determination of Mercury(II) on A Centrifugal Microfluidic Device Using Ionic Liquid Dispersive Liquid−Liquid Microextraction

**DOI:** 10.3390/mi10080523

**Published:** 2019-08-08

**Authors:** Yun Hui, Yujia Liu, William C. Tang, Dian Song, Marc Madou, Shanhong Xia, Tianzhun Wu

**Affiliations:** 1 Shenzhen Institutes of Advanced Technology, Chinese Academy of Sciences, Shenzhen 518055, China; 2 Department of Mechanical & Aerospace Engineering, University of California, Irvine, CA 92697, USA; 3 Department of Biomedical Engineering, University of California, Irvine, CA 92697, USA; 4 Department of Chemical and Biomolecular Engineering, University of California, Irvine, CA 92697, USA; 5 Institute of Electronics, Chinese Academy of Sciences, Beijing 100190, China

**Keywords:** centrifugal microfluidics, dispersive liquid-liquid microextraction, ionic liquid, valves, mercury ion

## Abstract

An integrated centrifugal microfluidic device was developed to preconcentrate and detect hazardous mercury (II) in water with ionic liquid as environmentally friendly extractant. An automatically salt-controlled ionic liquid dispersive liquid–liquid microextraction on a centrifugal microfluidic device was designed, fabricated, and characterized. The entire liquid transport mixing and separation process was controlled by rotation speed, siphon valves, and capillary valves. Still frame images on the rotating device showed the process in detail, revealing the sequential steps of mixing, siphon priming, transportation between chambers, and phase separation. The preconcentration of red dye could be clearly observed with the naked eye. By combining fluorescence probe and microscopy techniques, the device was tested to determine ppb-level mercury (II) in water, and was found to exhibit good linearity and low detection limit.

## 1. Introduction

Trace mercury(II) (Hg^2+^) contamination in water is highly hazardous to the environment in general and particularly to human health [1]. The United States Environmental Protection Agency (US-EPA) specifies that the maximum tolerable level of Hg^2+^ in potable water is 2 µg∙L^−1^ [2], while the World Health Organization (WHO) indicates a level of 6 µg∙L^−1^ as maximum. Conventional approaches to detect trace Hg^2+^ such as atomic fluorescence spectrometry (AFS) [3,4] and inductively coupled plasma mass spectrometry (ICP-MS) [5] require very expensive, complex, and bulky instruments. Recently, there have been research efforts in developing low-cost and efficient alternatives to achieve high selectivity and sensitivity, particularly by performing sample extraction and pre-concentration prior to detection [6,7,8].

Liquid−liquid extraction (LLE) is a classical sample preparation technique. Compared with LLE, dispersive liquid-liquid microextraction (DLLME) is advantageous because of the simplicity in its operations, high enrichment factor, and small amount of solvents requirement [9]. As a result, DLLME has been widely used for the determination of trace-concentration substances [10]. Ionic liquids (ILs), as environmentally friendly extractants, play increasingly important roles in extracting a large amount of bioactive compounds [11]. In our previous study, IL-DLLME was employed to highly preconcentrate ultra-trace mercury(II) followed by selective fluorescence detection (FD) to demonstrate very low detection limit [12]. However, manual operation of IL-DLLME was labor-intensive, instrument-dependent, time-consuming, and error-prone.

Microfluidic devices offer attractive alternatives for sample separation, detection, and analysis because of their inherent cost-effective fabrication process, requiring small sample size due to miniaturization, and amenable to automation by integrating multiple functions on the same platform [13,14]. Centrifugal microfluidics have been extensively researched to perform liquid transports and processing within the integrated platform using centrifugal force for flow control. A typical centrifugal microfluidic platform is usually integrated with multiple fluidic functions, such as valving, mixing, metering, separating, and detecting [15,16,17]. Hence, there is strong motivation to implement sample pretreatment on centrifugal microfluidic devices, also known as “Lab-on-a-disc” [18]. For example, Alexei et al. utilized pneumatic pumping for a multi-cycle LLE process on a centrifugal microfluidic device [19]. Also, Lidia et al. performed LLE on a centrifugal microfluidic device with extraction efficiency matching that of conventional batch extraction [20]. 

To the best of our knowledge, there have been no reports of performing IL-DLLME with a centrifugal microfluidic device, especially considering the large volume ratio of sample to IL. Based on the salting-out effect of *N*-octylpyridinium tetrafluoroborate IL [21] and IL-DLLME-FD method [12], we successfully performed salt-controlled (SC) IL-DLLME-FD method for the detection of Hg^2+^ with a centrifugal microfluidic device. Red dye was used to validate this method, which allowed visual examination with the naked eye of the high degree of enrichment. Encouraging analytical performance in determining trace concentration of Hg^2+^ was demonstrated, with further potential advantages in miniaturization, integration, and cost-effective fabrication.

## 2. Materials and Experimental

### 2.1. Reagents

Sodium perchlorate (NaClO_4_), mercuric chloride (HgCl_2_), sodium hydroxide (NaOH), and acetonitrile (ACN) were purchased from Sigma-Aldrich (St. Louis, MI, USA). *N*-octylpyridinium tetrafluoroborate ([OPy]^+^[BF4]^–^) was obtained from Shanghai Chengjie Chemical Co., Ltd. (Shanghai, China) Red dye was purchased from California Concepts on Amazon. The specific fluorescence probe of PTR (para-position-tetraphenylethenerhodamine derivative) for Hg^2+^ was provided by Benzhong Tang group (Hong Kong, China). Deionized water was used throughout this work.

### 2.2. Disc Fabrication

**Figure 1:**
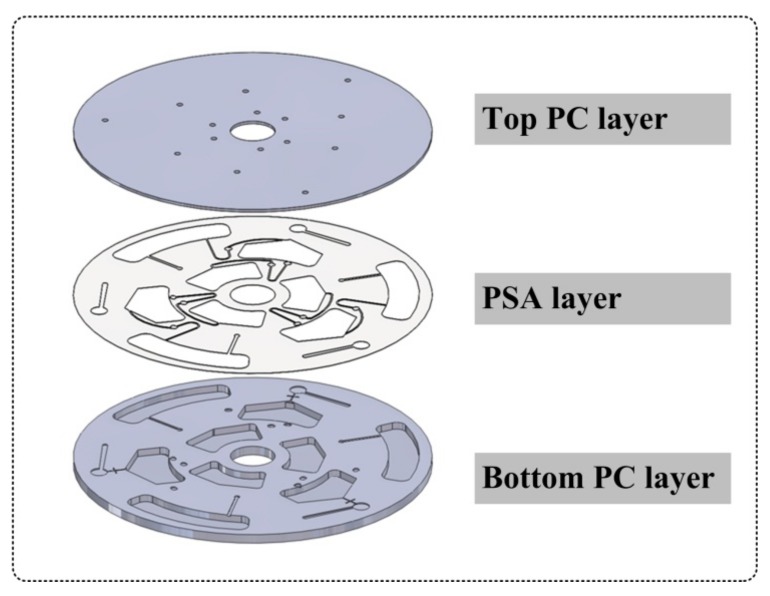
3D rendering of the three-layer structure of the centrifugal microfluidic disc.

Figure 1 shows the 3-D rendering of the three-layer disc (120 mm in diameter), which was designed with SolidWorks 2017 (SolidWorks Corp, Concord, MA, USA). The disc consisted of a top and a bottom layers of clear polycarbonate (PC) (McMaster, Carr, CA, USA) that were fabricated with computer-numerical control (CNC) machine (QuickCircuit 5000, T-tech Inc., Norcross, GA, USA). These two layers of PC were carefully aligned and bonded with double-sided pressure sensitive adhesive (PSA) tape (DFM 200 Clear V-95, FLEXcon, Spencer, MA, USA). The PSA layer was patterned with a cutting plotter (Silhouette America, Inc., Silhouette CameoTM, Lindon, UT, USA). During assembly, a fused silica capillary tubing (Polymicro Technologies, Phoenix, AZ, USA) was carefully embedded inside the channel with epoxy glue (Gorilla Glue, the Gorilla Glue Co., Cincinnati, OH, USA), as described by Angela, et al. [22]. Finally, these three layers were pressed firmly with the rolling machine. Note that there were three identical devices on the disc, each occupying 120° of the disc surface.

### 2.3. Experimental Setup

**Figure 2:**
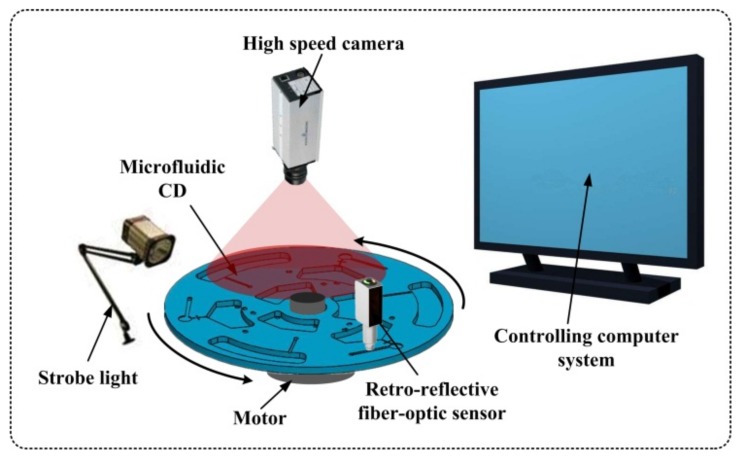
Illustration of the spinning test system, consisting of a computer controller connected to a stroboscopic imaging system over a servo-controlled spinning platform. The retro-reflective fiber-optic sensor was used to detect disc position to allow strobe synchronization.

As shown in Figure 2, the spinning test system consisted of a controlling computer system connected to a stroboscopic imaging system to visualize the sequential steps of the entire fluidic process on the disc. The imaging system consisted of a high-speed camera (Basler A301bc, 640 × 480 pixels, 80 fps maximum with 5× zoom lens), a strobe light (PerkinElmer MVS-4200, 6-µs duration), and a retro-reflective fiber-optic sensor (Banner D10 Expert Fiber-Optic Sensor). A servo motor (Pacific Scientific Servo Motor) with a motor controller (PAC SCI Programmable Servo Drive) was used to drive the device spinning at pre-programmed rotation speeds. With the strobe light synchronized with the servo motor through the retro-reflective fiber-optic sensor at three flashes per revolution, the camera captured one still frame image of each of the three identical devices on every complete revolution for fluidic movement observations.

### 2.4. Disc Design

**Figure 3:**
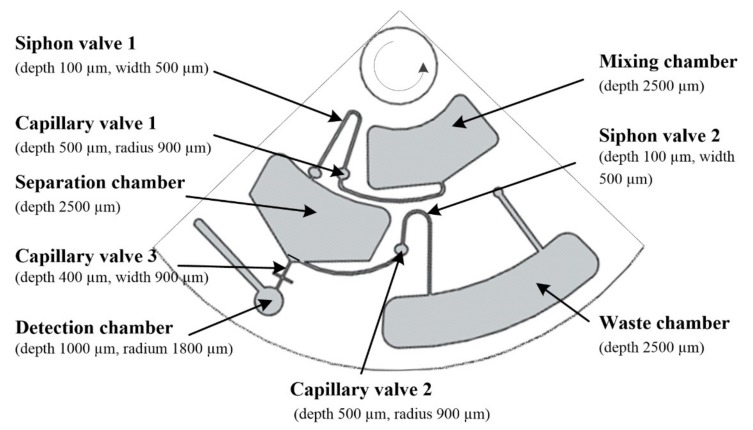
Layout of one of the three identical devices on the ionic liquid-dispersive liquid–liquid microextraction (IL-DLLME) disc.

There were three identical devices on the IL-DLLME disc, and one of them is shown in Figure 3. The total volume of the mixing, separation, and waste chamber was about 800 µL, which was sufficient to accommodate 600 µL of sample solution. 

**Figure 4:**
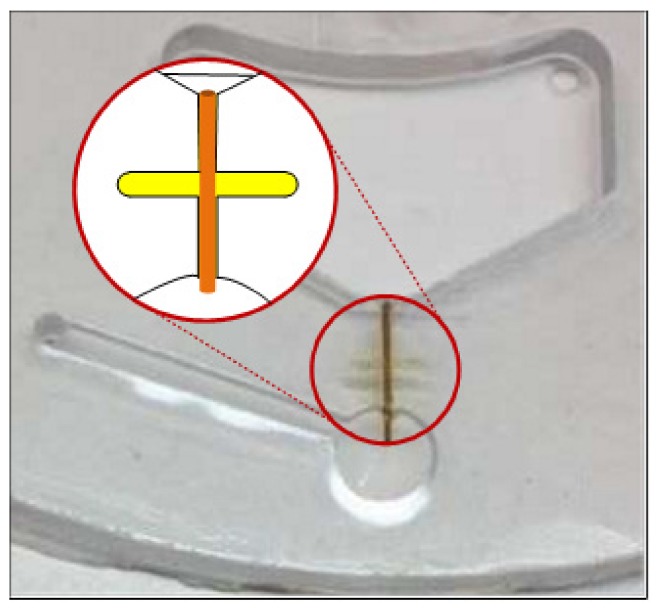
Photomicrograph of a sealed capillary valve embedded inside an assembled disc.

Effective valving techniques were the key to keeping the liquid sample isolated from the rest of the system during various operations [23]. One such valving mechanism was based on surface tension force inside a capillary channel that was designed to normally prevent fluidic flow between two adjacent chambers. At a specific spin speed designated as the burst frequency, the centrifugal force would overcome the surface tension force and the fluid would be forced through the channel. The siphon crests in the disc were closer to the disc center than the upper edge of the mixing chamber and separation chamber. Capillary valves connected to the siphon valves were designed to prevent hydrophilic siphon priming during sample solution mixing with IL. The fused silica capillary tube (with the outer diameter of 363 μm) was pretreated with 0.1 M NaOH and ethyl alcohol, then dried with air and cut to 6 mm long. After dipping into IL, the capillary tubes were glued into the milled channels (400 µm wide by 400 µm deep) in the bottom PC layer (Figure 4). The patterned PSA film was then carefully aligned on the upper surface of the bottom PC layer. Finally, the top PC layer was aligned and pressed onto the PSA. The three-layer sandwich was then permanently bonded with the pressure roller. This low-cost disc was designed to be disposable after single use to promote good test repeatability with a new device for every test.

Due to the high viscosity of IL and sufficient assembly accuracy of the three-layer disc, Capillary Valve 3 could be designed to sustain a burst frequency higher than the rotation frequency driving the supernatant out from the separation chamber. The Burst speed of fused silica capillary tubes with different inner diameter was tested. The results were listed in Table 1. The capillary tubes with the smallest inner diameter (24 µm) could sustain the highest rotation speed but suffered from reproducibility problems. The tests showed that capillary tubes with 51 µm inner diameter could be sufficient to serve as the multiple valves for all the sequential fluid processing.

## 3. Results and Discussion

### 3.1. Salt-controlled Ionic Liquid Dispersive Liquid-liquid Microextraction (SC-IL-DLLME) Dye Solution

**Figure 5:**
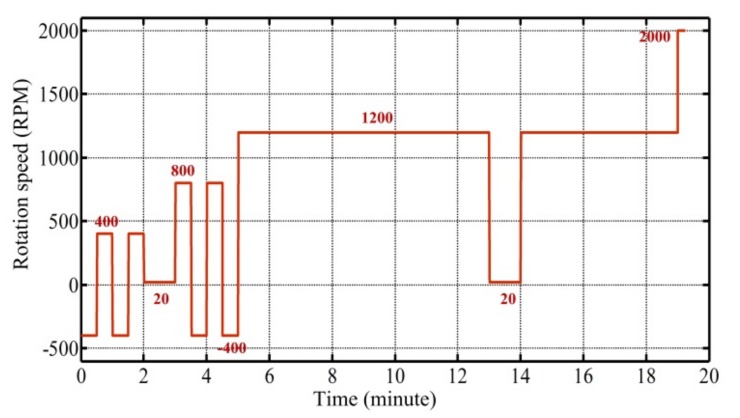
Spinning protocol in one salt-controlled ionic liquid dispersive liquid-liquid microextraction (SC-IL-DLLME) process.

**Figure 6:**
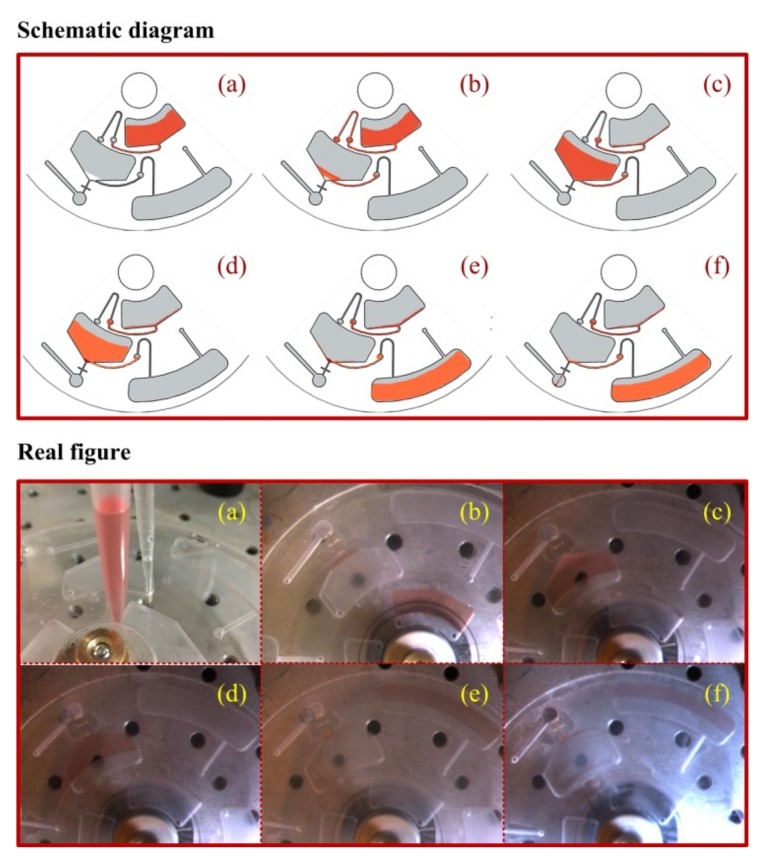
(**Top**) Schematics of salt-controlled ionic liquid dispersive liquid-liquid microextraction (SC-IL-DLLME) dye solution in operation (ionic liquid, white; NaClO_4_ solution, yellow). (**Bottom**) Corresponding experimental stroboscopic images of SC-IL-DLLME dye solution on the disc (ionic liquid, colorless; NaClO_4_ solution, colorless). The different panels are: (**a**) loading ionic liquid (IL), NaClO_4_ and dye (red), mixing IL and dye; (**b**) priming Siphon Valve 1, transporting from the mixing chamber to the separation chamber, (**c**) mixing IL and NaClO_4_, dispersing IL micro droplets; (**d**) centrifugation and depositing IL in the separation chamber, (**e**) priming Siphon Valve 2, transporting supernatant (pink) from the separation chamber to the waste chamber, and (**f**) bursting Capillary Valve 3, transporting enriched IL (dark red) phase from the separation chamber to the detection chamber.

Figure 5 shows the protocol for rotational speed control of the centrifugal disc in one SC-IL-DLLME process. It was found that rotation speeds of ±400 RPM and +800/−400 RPM in oscillation mode facilitated the mixing process. The acceleration or deceleration rates were experimentally determined and were all set at 1000 RPM·s^−1^. Figure 6 is a series of stroboscopic images obtained for the proof-of-concept fluid process experiment based on the protocol shown in Figure 5.

Initially, 3.5 µL IL and 600 µL red dye solution were respectively loaded into the mixing chamber at room temperature. It had been calculated that 3.5 µL IL could be dissolved in 600 µL aqueous solution at its saturation concentration, as shown in Figure S1 (in Supplementary Materials). A volume of 30 µL of 100 g·L^−1^ NaClO_4_ solution was loaded into the separation chamber (Figure 6a). Under moderate-speed rotation in both clockwise and counterclockwise directions, IL dissolved fully in the red dye solution forming a saturated solution. When the disc was spun at these speeds, the centrifugal force and 400 µm-deep capillary valve prevented the liquid from moving past the siphon crest. When the spinning speed decreased to a low enough speed, the capillary force overcame the centrifugal force, and surface tension pulled the meniscus over the crest [24], bringing the liquid through the hydrophilic siphon channel. Figure 6b showed the priming of Siphon Valve 1 and fluid flowing from the mixing chamber. The rotational speed was then increased in order to facilitate fluid transfer into the separation chamber. Subsequently, the homogeneous solution was fully mixed with NaClO_4_ solution in oscillation mode. Over-saturated IL was then forced to precipitate in a dispersive liquid phase upon contacting NaClO_4_ solution due to its high density. IL-water micro emulsion was instantly developed (Figure 6c).

After 8-minute spinning at 1200 RPM, the IL phase was deposited at the bottom, as shown in Figure 6d. The capillary tube was designed to overreach into the detection chamber, allowing sedimented IL to block fluidic flow and supernatant to empty through Siphon Valve 2. Then the rotation speed was decreased to 20 RPM, priming Siphon Valve 2 and allowing fluid to gradually flow from the separation chamber to the waste chamber (Figure 6e). The threshold burst rotation speed of the capillary valve could be up to 2000 RPM due to the high viscosity of IL. Figure 6f showed a line of IL in deep red, which was the result of enriched red dye. Finally, it was estimated that 2 µL IL with highly concentrated dye was collected in the detection chamber. This result was consistent with our previous investigation on the impact of NaClO_4_ on the solubility of IL in water [21]. In comparison to IL-DLLME in conventional approaches [12], the volume of the sample was reduced by two orders of magnitude and operation time was reduced to 20 min. 

### 3.2. Salt-controlled Ionic Liquid Dispersive Liquid-liquid Microextraction Fluorescence Detection (SC-IL-DLLME-FD) Method for Mercury Analysis

**Figure 7:**
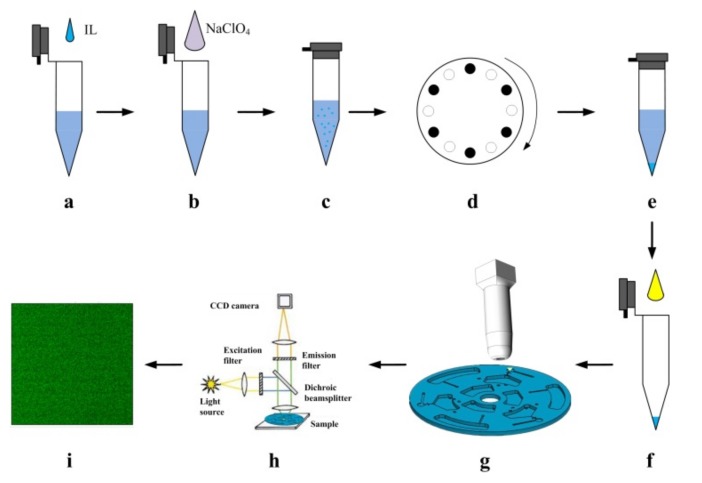
Conceptual illustration of the salt-controlled ionic liquid dispersive liquid-liquid microextraction fluorescence detection (SC-IL-DLLME-FD) process steps: (**a**) The mercury solution dissolved ionic liquid (IL), achieving saturated IL aqueous solution. (**b**) NaClO_4_ was added. (**c**) IL micro-droplets suspended in the emulsion. (**d**) Centrifugation. (**e**) Separation. (**f**) IL phase was mixed with acetonitrile (ACN) and aqueous solution containing para-position tetraphenylethenerhodamine (PTR) probe. (**g**) Reaction at 55 °C for one hour. (**h**) Mercury ion was quantified through fluorescence microscope. (**i**) Resulting fluorescent images.

**Figure 8:**
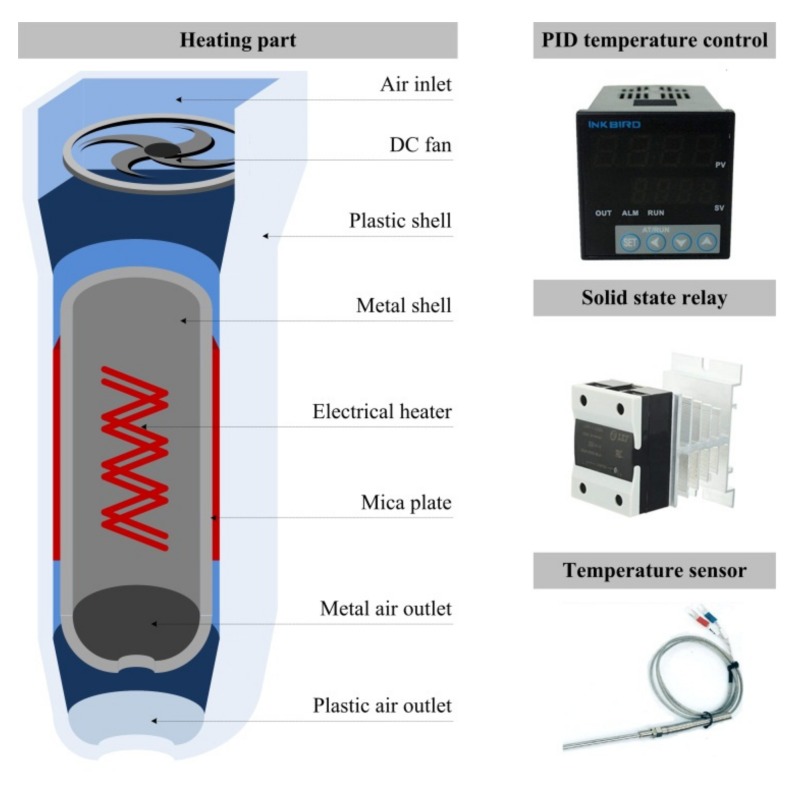
Schematics of the custom-built heating apparatus.

The entire procedure of SC-IL-DLLME-FD method for the determination of Hg^2+^ is conceptually illustrated in Figure 7. Thereinto, mixing chamber and separation chamber are replaced with centrifugal tubes for conceptual visualization. A custom-built heating apparatus was used to heat the solution in the detection chamber as shown in Figure 8. Although the fluorescence (FL) intensity grew as the heating time was increased (Figure S2, in Supplementary Materials), heating time was shortened to one hour for practical purposes. Two-photon fluorescence microscope and Image J software were used to obtain and analyze the FL intensity from mercury solutions at different concentrations. The optimum excitation wavelength of PTR probe was 345 nm as shown in Figure S3 (in Supplementary Materials). As a result, the excitation wavelength was set at 690 nm for two-photon fluorescence microscope. The remaining detection conditions were based on the experimental conditions in our previous study [12], the fluorescence images of 10 µM PTR probe and Hg^2+^ in different concentrations were collected in the detection solutions (approximately 2 µL IL, 1.8 µL ACN, and 4.2 µL water).

**Figure 9:**
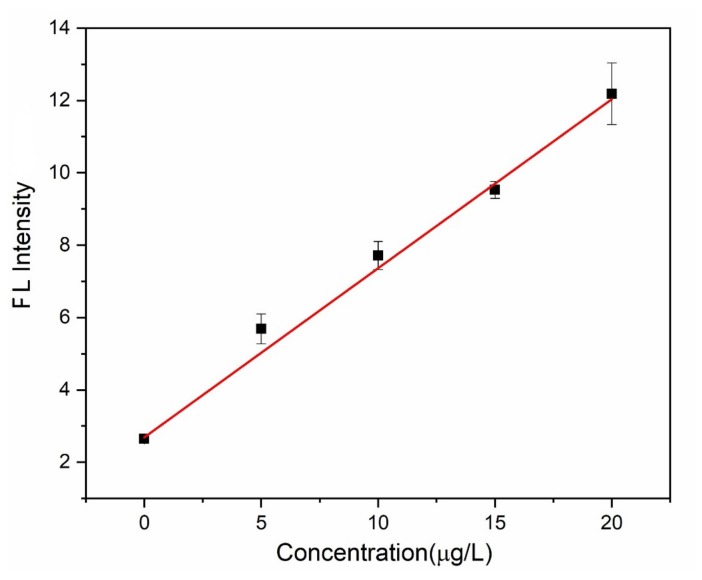
The fluorescence (FL) intensity as a function of the concentration of Hg^2+^.

As is shown in Figure 9, the prototype was tested with 0~20 µg∙L^−1^ Hg^2+^ solutions in deionized water, and good linearity (linear regression coefficient of R^2^ = 0.9953) was achieved within that range. Table 2 summarized the analytical performances obtained by the SC-IL-DLLME-FD method. According to the IUPAC recommendation [25], the limit of detection (LOD) and the limit of quantitation (LOQ) were calculated as the concentration yielding a peak area equal to the blank signal plus 3- and 10-times the blank standard deviation, respectively. Thus LOD was estimated to be 0.753 μg∙L^−1^, and LOQ was estimated to be 2.51 μg∙L^–1^, which satisfied the threshold limit established by WHO. Also, the LOD was much lower than the limit of 2 μg∙L^–1^ set by US-EPA, while the LOQ was very close.

## 4. Conclusions

In the work, an automated centrifugal microfluidic device was successfully demonstrated for the first time for performing whole salt-controlled ionic liquid dispersive liquid-liquid microextraction process. The experimental results confirmed that the fabricated disc significantly enriched red dye, demonstrating the feasibility of a viable alternative to conventional preconcentration and separation system. This novel SC-IL-DLLME disc was also successfully used for the determination of hazardous Hg^2+^, and the LOD was much lower than the EPA limit of Hg^2+^ ions in drinking water. Future work will address further improvement for the automation and integration in portable analytical instrumentations for environmental monitoring.

## Figures and Tables

**Table 1:** Burst frequency of capillary tubes with different inner diameters for ionic liquid (IL).

Inner Diameter	24 μm	51 μm	74 μm
Burst Frequency	2800~3500 RPM	1400~1800 RPM	800~1000 RPM

**Table 2:** Analytical performance of the disc for the detection of Hg^2+^.

Analytical Performance	Lab on the Disc ^1^
Slope	0.46751 ± 0.018
Intercept	2.68511 ± 0.133
Linear range	0~20 μg∙L^–1^
R^2^	0.9953
LOD (S/N = 3)	0.753 μg∙L^–1^

^1^ Calibration levels (n = 5) and Mean ± standard deviation (n = 3).

## Supplementary Materials

The following are available online at https://www.mdpi.com/2072-666X/10/8/523/s1, Figure S1: Influence of NaClO4 on the solubility of [OPy]+[BF4]– in water at room temperature, Figure S2: The fluorescence (FL) intensity of 10 µM PTR and 10 µM mercury solutions with 25% ionic liquid, water fraction of 70%, in water bath (55 °C), Figure S3: The fluorescence (FL) intensity at 589.2 nm under different excitation wavelengths.
