# Supplementary material for: Determination of Mercury(II) on A Centrifugal Microfluidic Device Using Ionic Liquid Dispersive Liquid−Liquid Microextraction
Source: Micromachines (Basel). 2019 Aug 8;10(8):523. doi: 10.3390/mi10080523 (PMC6723164; doi:10.3390/mi10080523)
Supplement: Supplementary file 1 [file micromachines-10-00523-s001.pdf]

Supplementary Material: Determination of Mercury (Ⅱ) on a Centrifugal Microfluidic Device Using Ionic Liquid Dispersive Liquid–Liquid Microextraction

Yun Hui [1], Yujia Liu [2], William C. Tang [3], Dian Song [4], Marc Madou [2], Shanhong Xia [5] and Tianzhun Wu [1,*]

1. Shenzhen Institutes of Advanced Technology, Chinese Academy of Sciences, Shenzhen, 518055, China
2. Department of Mechanical & Aerospace Engineering, University of California, Irvine, CA, 92697, USA
3. Department of Biomedical Engineering, University of California, Irvine, CA, 92697, USA
4. Department of Chemical and Biomolecular Engineering, University of California, Irvine, CA, 92697, USA
5. Institute of Electronics, Chinese Academy of Sciences, Beijing, 100190, China

* Correspondence: tz.wu@siat.ac.cn; Tel.: +86-755-8639-2339

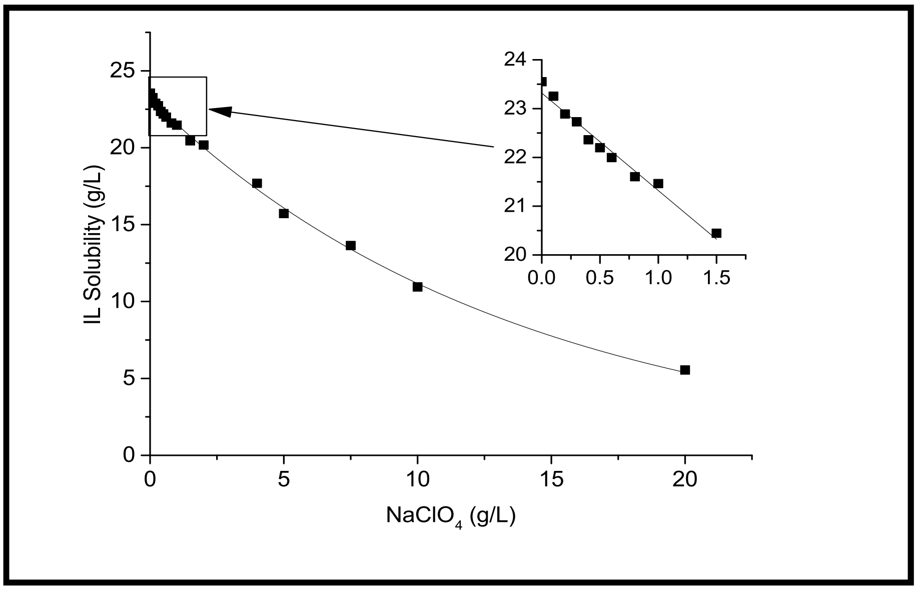

Figure S1. Influence of $NaClO_4$ on the solubility of $[OPy]^+[BF4]^-$ in water at room temperature.

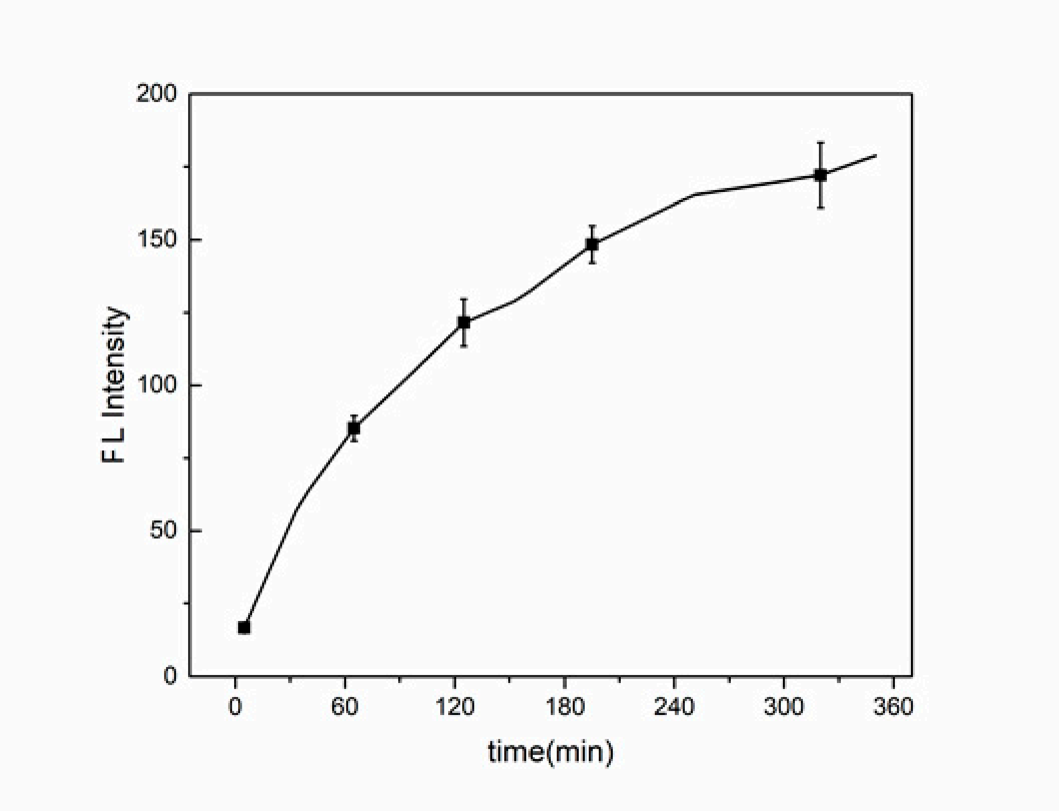

Figure S2. The FL intensity of 10 μM PTR and 10 μM mercury solutions with 25% IL, water fraction of 70%, in water bath (55℃).

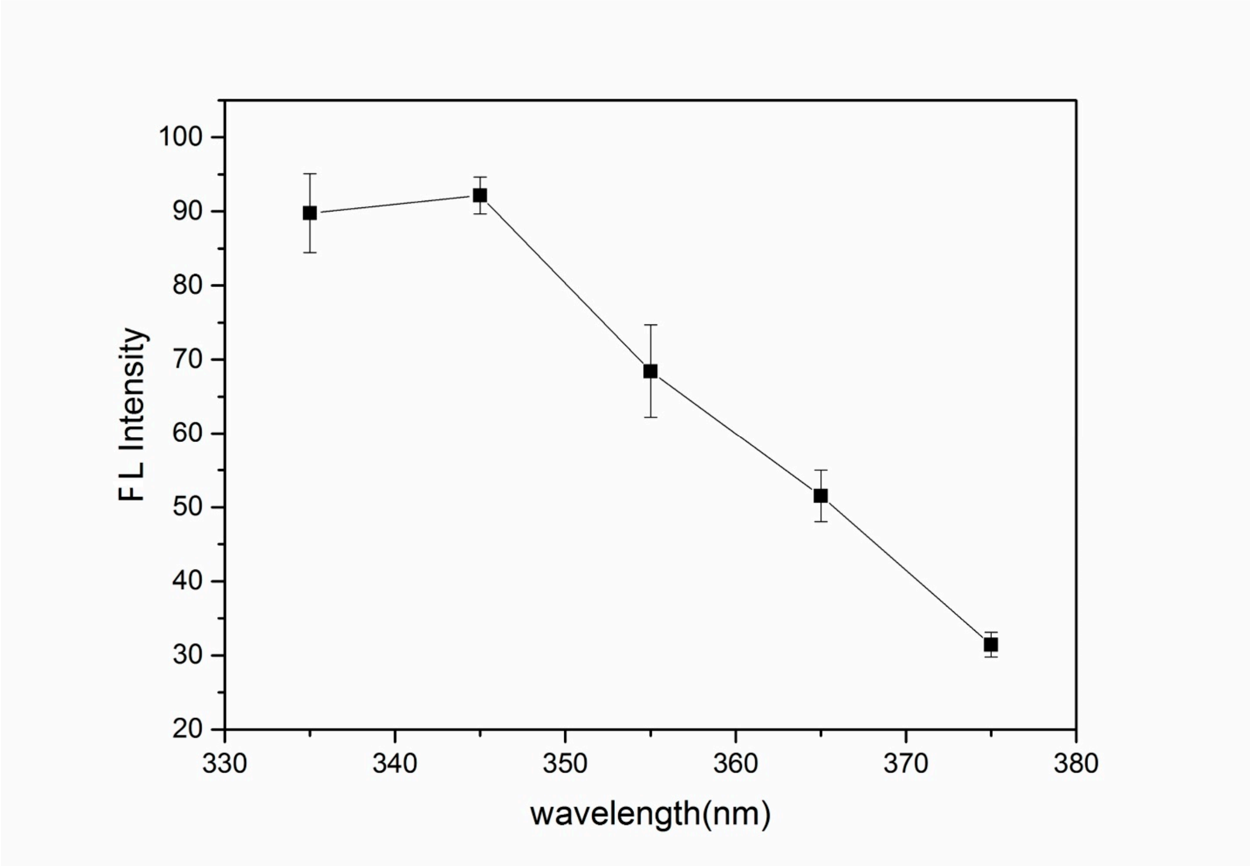

Figure S3. The fluorescence (FL) intensity at 589.2 nm under different excitation wavelengths.
